# Extensive Homoplasy but No Evidence of Convergent Evolution of Repeat Numbers at MIRU Loci in Modern *Mycobacterium tuberculosis* Lineages

**DOI:** 10.3389/fpubh.2020.00455

**Published:** 2020-08-27

**Authors:** Alexander C. Outhred, Ulziijargal Gurjav, Peter Jelfs, Nadine McCallum, Qinning Wang, Grant A. Hill-Cawthorne, Ben J. Marais, Vitali Sintchenko

**Affiliations:** ^1^Marie Bashir Institute for Infectious Diseases and Biosecurity, The University of Sydney, Sydney, NSW, Australia; ^2^Children's Hospital at Westmead, Sydney, NSW, Australia; ^3^Center for Infectious Diseases and Microbiology—Public Health, Westmead Hospital, Sydney, NSW, Australia; ^4^Department of Microbiology and Immunology, Mongolian National University of Medical Sciences, Ulaanbaatar, Mongolia; ^5^NSW Mycobacterium Reference Laboratory, Center for Infectious Diseases and Microbiology Laboratory Services, Institute of Clinical Pathology and Medical Research—NSW Health Pathology, Sydney, NSW, Australia; ^6^Deep Seq Lab, Queen's Medical Center, University of Nottingham, Nottingham, United Kingdom; ^7^School of Public Health, University of Sydney, Sydney, NSW, Australia

**Keywords:** *Mycobacterium tuberculosis*, homoplasy, convergent evolution, MIRU, VNTR, phylogeny, sensitivity, specificity

## Abstract

More human deaths have been attributable to *Mycobacterium tuberculosis* than any other pathogen, and the epidemic is sustained by ongoing transmission. Various typing schemes have been developed to identify strain-specific differences and track transmission dynamics in affected communities, with recent introduction of whole genome sequencing providing the most accurate assessment. Mycobacterial interspersed repetitive unit (MIRU) typing is a family of variable number tandem repeat schemes that have been widely used to study the molecular epidemiology of *M. tuberculosis*. MIRU typing was used in most well-resourced settings to perform routine molecular epidemiology. Instances of MIRU homoplasy have been observed in comparison with sequence-based phylogenies, limiting its discriminatory value. A fundamental question is whether the observed homoplasy arises purely through stochastic processes, or whether there is evidence of natural selection. We compared repeat numbers at 24 MIRU loci with a whole genome sequence-based phylogeny of 245 isolates representing three modern *M. tuberculosis* lineages. This analysis demonstrated extensive homoplasy of repeat numbers, but did not detect any evidence of natural selection of repeat numbers, at least since the ancestral branching of the three modern lineages of *M. tuberculosis*. In addition, we observed good sensitivity but poor specificity and positive predictive values of MIRU-24 to detect clusters of recent transmission, as defined by whole-genome single nucleotide polymorphism analysis. These findings provide mechanistic insight, and support a transition away from VNTR-based typing toward sequence-based typing schemes for both research and public health purposes.

## Introduction

Mycobacterial Interspersed Repetitive Unit (MIRU) typing (1–3) has been widely used to identify epidemiological clusters and potential laboratory contamination incidents involving *Mycobacterium tuberculosis* complex. MIRU is based on measuring the length of variable number tandem repeat (VNTR) loci in the *M. tuberculosis* genome, which is conceptually the same as subtyping of other bacterial genera by multiple locus VNTR analysis (MLVA). Different sets of loci can be used for analysis, and common variants include 12-locus MIRU (i.e., MIRU-12), 15- and 24-locus MIRU (MIRU-24). The increased discrimination provided by a greater number of informative loci needs to be balanced against the cost and complexity of the test. MIRU-24 is widely regarded as a well-standardized strain typing method that provides adequate discrimination for the majority of *M. tuberculosis* strains.

Australia has a low incidence of tuberculosis (5–6/100,000 population), that can be sub-classified into pre-elimination rates among people of non-Indigenous origin born in Australia (~1/100,000 population), with higher rates (~20/100,000 population) in individuals born overseas (4). A strong majority (>80%) of tuberculosis cases are overseas-born, implying a very low rate of *M. tuberculosis* transmission within Australia. By comparison, incidence rates of tuberculosis in England are somewhat higher but broadly similar [in overseas-born individuals, ~40/100,000, and in local-born individuals, ~3/100,000 (5)]. To help investigate potential local transmission, the largest MIRU-24 clusters identified in the state of New South Wales (NSW) over a 5-year period (2009–2013) were subjected to whole-genome sequencing (6). These MIRU-24 clusters were the two largest Lineage 1 (“Indo-Oceanic” or EAI) and two largest Lineage 2 (East Asian or “Beijing”) clusters. Excluding three isolates related to laboratory cross-contamination, whole-genome sequencing reduced the estimated number of secondary cases from 23 isolates in four transmission chains to one isolate in one chain. These results, along with other recent reports (7–9), reveal important limitations in the value of MIRU-24 to identify likely transmission events and guide public health responses in low-transmission settings where the vast majority of *M. tuberculosis* strains are imported. Although MIRU-24 has been widely used in research and public health contexts to help understand *M. tuberculosis* transmission dynamics and to characterize relapse vs. reinfection events, it is limited by false-positive and false-negative clustering. Relaxing the cluster definition to include isolates with differences at one or two MIRU loci has been widely used to reduce false-negative clustering, but worsens false-positive clustering. Mathematical modeling suggests that homoplasy of repeat loci can partially explain false-positive MIRU clustering (10).

Homoplasy occurs when an identical characteristic arises in unrelated clades; in other words, a trait is shared for reasons other than descent from a common ancestor. Homoplasy can arise as a result of horizontal gene transfer, but this is rare in *M. tuberculosis*. More typically, homoplasy arises through stochastic processes, and can also be amplified by natural selection (convergent evolution, where certain traits have higher fitness). In *M. tuberculosis*, selection of mutations conferring drug resistance provides clear evidence of homoplasy through convergent evolution; for example, the p.Ser315Thr mutation in the *katG* gene has evolved numerous times in unrelated clades under isoniazid-induced selective pressure (11, 12). The Retention Index gives a value between 0 and 1 representing the homoplasy of a trait with reference to a phylogenetic tree; this index is the complement of the fraction of possible homoplasy that is present in the tree, so that a value of zero indicates maximal homoplasy, and a value of one indicates absence of homoplasy (13).

The discriminatory power of strain typing methods is best understood by considering mathematical indices of diversity, such as the Simpson index (14, 15). *M. tuberculosis* isolates derived from settings with low rates of *M. tuberculosis* transmission where unlinked cases of tuberculosis dominate, such as Australia and the United Kingdom, capture diverse *M. tuberculosis* sub-lineages. A diverse phylogeny provides an opportunity to assess the potential for MIRU to capture the underlying diversity, to measure the reduction in observed diversity resulting from MIRU homoplasy, and to detect evolutionary signals contributing toward MIRU homoplasy.

MIRU homoplasy has been observed numerous times previously (16–18) and simulated with a detailed model (17). The first thorough sequence-based assessment graphically depicted homoplasy in a multi-locus sequence analysis phylogeny of 97 Lineage 1–6 strains, and compared the maximum likelihoods of sequence-based and VNTR-based trees (16). In another instance comparing sequence- and VNTR-based phylogenies, a VNTR-based minimum spanning tree was accepted as the reference phylogeny and homoplasy of a SNP and an *IS6110* insertion site was described (19). In some reports it has been stated that the observed homoplasy is the result of convergent evolution (16, 18), however there has been no previous quantification of homoplasy across repeat loci in reference to a whole genome phylogeny, and no formal testing for a phylogenetic signal of convergent evolution as a driver of MIRU homoplasy.

The aim of this study was to quantify the extent and impact of MIRU locus homoplasy in modern phylogenetic lineages of *M. tuberculosis* on the potential public health utility of MIRU typing, and to test whether homoplasy can be attributed to convergent evolution. To test for a phylogenetic signal, we treated the number of repeats at MIRU loci as a trait or characteristic undergoing evolution along branches of the phylogenetic tree, regarding number of repeats at a MIRU locus as an ordinal trait. Ordinal traits are commonly treated as “pseudo-continuous” traits (20, 21); treating repeat number as a pseudo-continuous trait enables the use of Brownian motion and Ornstein–Uhlenbeck models of evolution along branches (22, 23). Brownian motion is the standard model of continuous trait evolution along branches by purely stochastic processes in the absence of natural selection. Ornstein-Uhlenbeck models add a parameter to the Brownian motion model that enables estimation of selective pressure in a manner that is biologically plausible, and can enable the detection of changes in selective pressure in different clades of a tree (24, 25). Most alternate measures to detect phylogenetic signal, such as Pagel's λ, have more difficult interpretation as there is no underlying biological basis (e.g., [*http://www.carlboettiger.info/2013/10/11/is-it-time-to-retire-pagels-lambda.html*], accessed 16-Nov-2018).

## Materials and Methods

### Selection of Isolates

*M. tuberculosis* strains from patients diagnosed between 2009 and 2013 and characterized by the NSW Mycobacterium Reference Laboratory (MRL) underwent MIRU-24 typing as described previously (6, 26, 27); a subset of 30 isolates was sequenced using the Ion Torrent platform and the Ion 318 kit (Thermo Fisher). A literature search was performed for publications reporting *M. tuberculosis* isolates with publicly available whole-genome sequencing data and MIRU-24 data; two papers were identified (28, 29). Merker et al. (29) generated a worldwide phylogenetic analysis of Beijing strains (Lineage 2). Walker et al. (28) analyzed UK strains, including MIRU-matched community clusters of *M. tuberculosis*. We combined these publicly available datasets with findings from the two largest MIRU-24 Beijing strain clusters identified at the NSW MRL from 2009 to 2013 (6).

**Figure 1:**
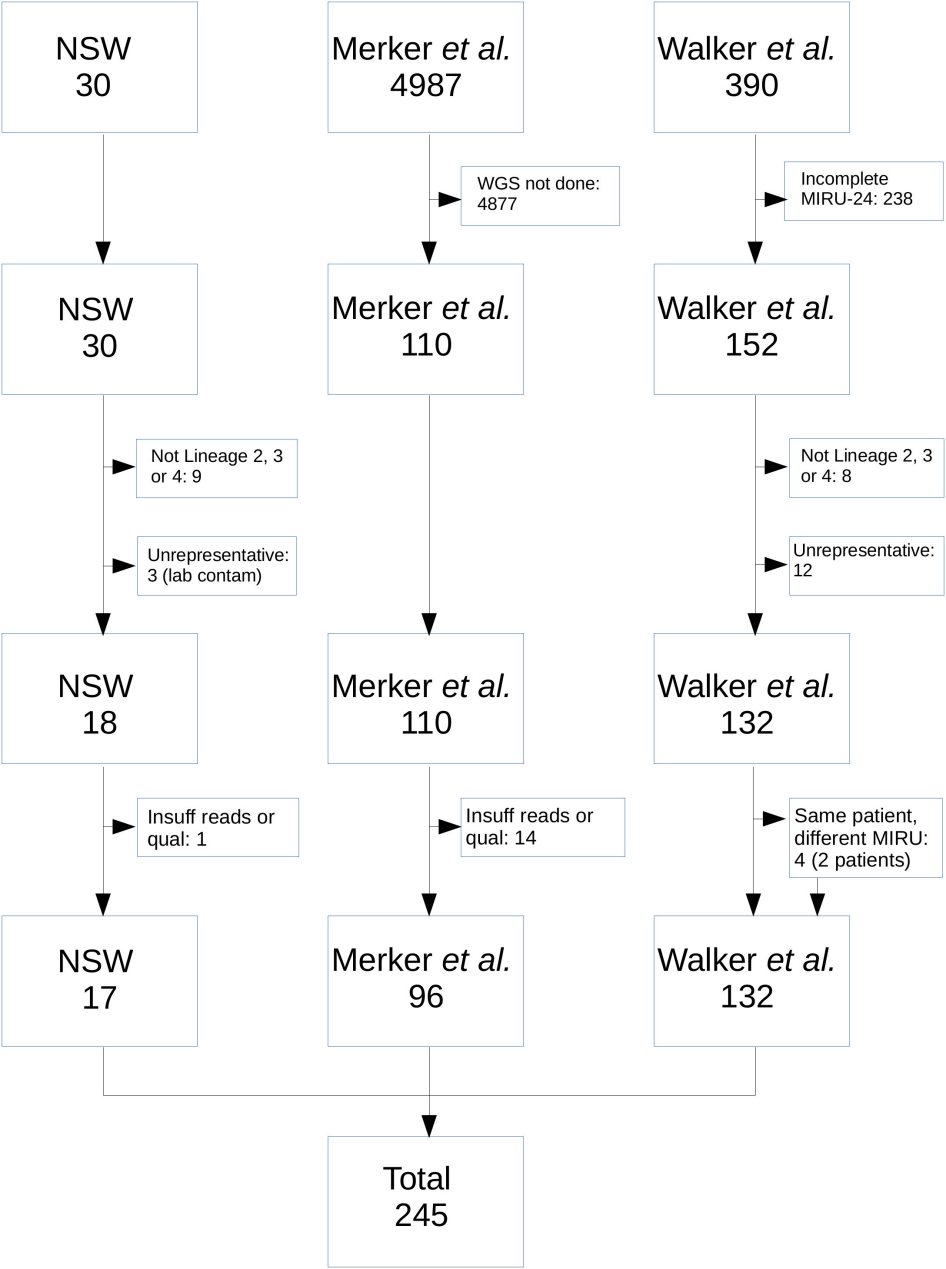
Flow diagram of *M. tuberculosis* isolates from the NSW, Merker, and Walker collections included in the analysis. From NSW, nine isolates were excluded as they were not from Lineage 2, 3, or 4; 3 were excluded as they arose as a result of probable laboratory cross-contamination incidents in referring laboratories; 1 was excluded due to insufficient read number and quality. From the Merker collection, 4,877 were excluded because WGS was not performed; 14 were excluded due to insufficient number or quality of reads. From the Walker collection, 238 were excluded because MIRU-24 was not completely ascertained; 8 were excluded as they were not from Lineage 2, 3, or 4; 12 were excluded due to high *a priori* probability that isolates were identical (e.g., same patient and same MIRU-24). An additional four isolates from two patients in the Walker collection were retained in the analysis (i.e., same patient but different MIRU-24).

Figure 1 shows the selection process for community *M. tuberculosis* isolates eligible for inclusion. Isolates without corresponding WGS data and complete MIRU-24 were excluded. Isolates that were not members of Lineage 2, 3, or 4 were excluded, as the number with complete sequence and MIRU-24 data was too small for reliable analysis. Isolates with strong *a priori* basis to assume identity were excluded (e.g., suspected laboratory cross-contamination, or isolates with same MIRU-24 from the same patient; however neither community nor household MIRU-24 clusters were excluded). The 17 NSW MRL isolates that met all criteria for inclusion comprised one 8-member and one 9-member MIRU-24 cluster. The final set of isolates and their origin is shown in Supplementary Table S1.

### MIRU Typing of Isolates

Linked MIRU-24 and whole-genome sequencing data for isolates in these three datasets enabled a comparison of MIRU-24 evolution with the whole-genome sequencing phylogeny of a global strain collection. The order of MIRU loci adopted for the concatenated MIRU-24 string was 154, 580, 960, 1644, 2059, 2531, 2687, 2996, 3007, 3192, 4348, 802, 424, 577, 1955, 2163b, 2165, 2347, 2401, 2461, 3171, 3690, 4052, and 4156.

### Sequencing Data Analysis

FASTQ files corresponding to 110 eligible isolates from the Merker (29) collection were downloaded from the European Nucleotide Archive (ENA), together with MIRU-24 data in the supporting information. FASTQ files corresponding to 132 eligible isolates from the Walker (28) collection were downloaded from ENA, together with MIRU-24 data in the supporting information. Together with 18 isolates from NSW, FASTQ files from 260 isolates were trimmed of library and sequencing adaptors using *trimadap* [https://github.com/lh3/trimadap]. FASTQ files from the Ion Torrent platform, used to sequence clustered Lineage 2 strains identified in NSW, underwent quality-score-preserving error correction using *muffinec* (30). FASTQ files from Illumina platforms underwent error correction using *bfc* (31). A reference sequence for mapping was derived from NC_000962.3 by substituting gaps for repetitive elements (all elements annotated as PE/PPE/PGRS and *cysA* genes, insertion sequences, transposases and prophage components, replacing 6.3% of the H37Rv genome with gaps). Mapping of error-corrected reads against this reference sequence was performed using the *mem* algorithm of *bwa* (32). Duplicates were marked using *samblaster* (33). Libraries with mean genome coverage below 20 were excluded. Variants were called from a merged BAM file using *freebayes* (34), specifying a haploid genome and requiring 90% of reads to support each called variant. The resulting VCF file was filtered for variants that were high confidence [QUAL > 200 and ODDS > 19, where ODDS represents the marginal likelihood ratio of the called variant (34)], informative (each variant called for at least 1 sample but not for all samples), and categorized as a SNP (CIGAR = 1X). Variants were annotated using *snpEff* (35). SNPs within 150 nucleotides of the genes *gyrA, katG, pncA, rpoB, inhA, fabG1, ahpC, embA, embB, gid, eis, ndh, rpsA, rpsL, rrs*, and *tlyA* were considered potentially homoplasious (potentially selected through drug exposure) and were excluded from further analysis. The remaining SNP variants for each sample were patched into the reference genome using *vcf2fasta* [https://github.com/vcflib/vcflib].

A Bayesian inference phylogeny was generated from the concatenated alignment of 245 whole genomes using *mrbayes* (36), with the “mixed” model of nucleotide substitutions and a gamma distribution of substitution rates (approximated in four categories). Tip dates were not used, as it was assumed that time since the most recent common ancestor (MRCA) was much greater than the difference between *M. tuberculosis* isolation dates in the current study.

### Comparison of MIRU Data With Whole Genome Phylogeny

MIRU-24 and SNP-based phylogenies of *M. tuberculosis* were examined in order to explore potential homoplasy in MIRU loci, focusing on strains from Lineages 2, 3, and 4 of *M. tuberculosis*. The distance between genomes of *M. tuberculosis* was measured by patristic distance. Higher mean patristic distance (MPD) is associated with descent from more ancestral strains; in other words, for a leaf with high MPD the branches leading toward it are long with few near neighbors. Nearest taxon distance (NTD) is a property of leaves and refers to the total substitution distance between a leaf and its nearest neighbor. Calculation of these distances from the *mrbayes* tree was performed using *DendroPy* (37). Further statistical analysis of MIRU-24 parameters and the phylogenetic tree was performed using *R* modules *ape, bayou, phangorn, phylobase, phytools*, and *vegan* (25, 38–40). Phylogenetic trees were visualized using *R ggtree* (41).

A distance between isolates (NTD) of <10 substitutions was arbitrarily chosen as consistent with recent transmission; this is roughly equivalent to 14–32 years of bacterial evolution (or 7–16 years on each branch of a symmetrical pair) based on recent estimates of the *M. tuberculosis* molecular clock (28, 42–44), and hence does not necessarily indicate direct transmission between patients.

## Results

In total, we assessed the MIRU-24 profiles and SNP-based phylogeny of 245 isolates; 17 were derived from two MIRU-24 clusters identified in NSW Australia, 132 from the Walker collection of United Kingdom community MIRU-24 clusters, and 96 from a global collection of Lineage 2 strains, as shown in Figure 1. Fourteen of the 110 eligible isolates from the Merker collection were excluded from further analysis due to short read length (50 bp) or insufficient number of reads after *bfc* error correction. Four of 21 isolates from the NSW MRL were excluded from further analysis, one due to insufficient number of reads after *muffinec* error correction and three that represented likely laboratory cross-contamination. Of the 245 isolates, 118 (48%) were from Lineage 2, 35 (14%) from Lineage 3 and 92 (38%) from Lineage 4. From a total of 10,965 SNPs, 115 (1%) were excluded from analysis as potentially homoplasious (in genes where resistance-associated SNPs are believed to occur); 37 of these 115 SNPs were synonymous and 58 were present in more than one isolate. The median number of isolates possessing one of these 115 SNPs was two, suggesting that the impact of their exclusion on the phylogeny is minimal and that few of these 115 SNPs were truly homoplasious.

**Figure 2:**
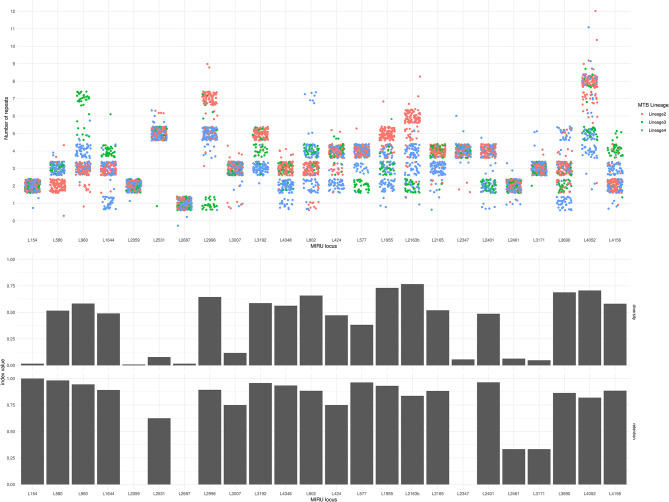
MIRU locus, number of repeats, diversity index, and retention index for 245 Lineage 2, 3, and 4 *M. tuberculosis* isolates. In the top panel, each circle represents the number of repeats (*y*-axis) at each MIRU locus (*x*-axis) of one of the 245 isolates. Jitter has been applied in horizontal and vertical axes to reduce overlap. Circles are color-coded by Lineage. The middle panel shows the Simpson diversity index of each MIRU locus (0 represents complete absence of diversity, and 1 represents complete diversity). The bottom panel shows the retention index of each MIRU locus (0 represents complete homoplasy, and 1 represents complete absence of homoplasy). Note that loci 2347 and 2687 have retention indices of zero, and locus 2059 has an undefined retention index due to only a single instance of variation in this phylogeny.

The Simpson diversity index for individual MIRU-24 loci ranged from 0.008 to 0.77 (median 0.50, with eight loci below 0.2). The Simpson index for the complete MIRU-24 was 0.96. In this dataset from lineages 2, 3, and 4, four loci could account for almost all of the total MIRU-24 diversity (Simpson index 0.94 using loci 1955, 2163b, 3690, and 4052). Other data transformations could preserve comparable diversity (e.g., Simpson index 0.92 for the sum total number of repeats across all 24 loci). Figure 2 shows the distribution of repeat numbers by MIRU locus, and shows the corresponding Simpson diversity and retention indexes. The number of repeats was variable in 10 or fewer of the 245 strains at 7 MIRU loci. For MIRU loci with Simpson diversity >0.1, the retention index (using the whole genome phylogeny as reference) ranged from 0.75 (locus 424) to 0.98 (locus 580). The four MIRU loci with highest Simpson diversity (1955, 2163b [QUB-116], 3690 [VNTR-52], and 4052 [QUB-26]) had retention indices of 0.93, 0.84, 0.86, and 0.82. The lower retention indices of loci 2163b and 4052 indicate a higher degree of homoplasy. In this Lineage 2, 3, and 4 dataset, MIRU locus 2163b had the highest Simpson diversity but one of the lowest retention indices. Locus 1955 had high diversity and also a relatively high retention index, but other loci were less informative.

**Figure 3:**
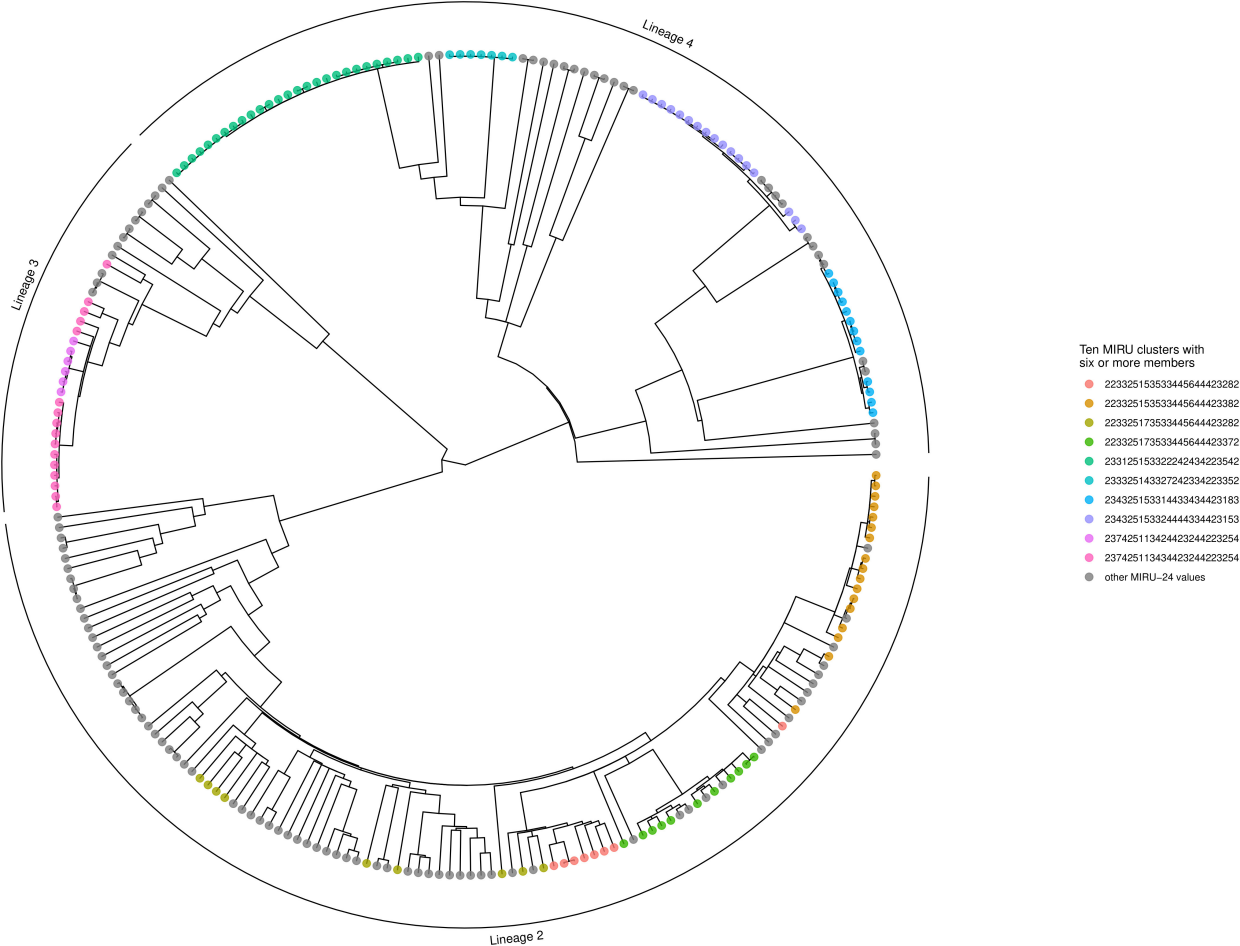
Phylogenetic tree showing MIRU clusters with six or more members. The phylogenetic tree is ultrametric and was generated using Bayesian inference of whole genomes with substituted SNPs (*n* = 10,850) and a uniform clock (scale not shown; the height of the tree from root to tips is ~410 substitutions). Segments of the tree corresponding to Lineages 2, 3, and 4 are labeled with an outer arc. Isolates corresponding to the 10 largest MIRU-24 clusters are labeled with colored circles at the tips. Instances of repeat number homoplasy that affect the complete MIRU-24 are present in Lineage 2. The tree was generated using *R ggtree* (41).

The Bayesian inference phylogeny, with tips color-coded according to membership of the 10 largest MIRU-24 clusters (containing six or more members), is shown as Figure 3 (a corresponding figure with tips labeled by sample name is available as Supplementary Image 1). The MPD across all tips of the tree was 604 substitutions. The mean nearest taxon distance (MNTD) was 52 substitutions. Capturing diversity of a phylogenetic tree in a single index (comparable with Simpson diversity for MIRU) is difficult, but summary values derived from the tree for each tip—MPD, NTD, and median taxon distance—produced Simpson indices of 0.99, 0.99, and 0.97, respectively; these summary values encompass a higher diversity than MIRU-24.

### MIRU-24 and SNP Clusters

One 8-member and one 9-member MIRU cluster among the 17 NSW isolates were identified using an identical MIRU-24 match as the cluster definition. From the Merker collection, one 17-member and one 11-member cluster were identified. In the Walker collection, there were one 27-member, one 17-member, one 16-member, one 13-member, one 7-member, and one 6-member clusters. The remaining 114 isolates were MIRU-24 singletons (68 isolates), pairs (22 isolates), or members of MIRU-24 clusters with three to five members (24 isolates). Extending a MIRU-24 cluster to include isolates with up to one mismatched locus produced a cluster with 50 strains; including up to two mismatched loci (28) produced a cluster with 77 strains. These relaxed cluster definitions were considered unhelpful and not analyzed further.

The distance tree revealed 138 isolates that were <10 substitutions from their nearest taxon, consistent with a recent shared ancestor (shown in Supplementary Image 2). These were distributed in 30 SNP-clusters; 30 isolates in 9 SNP-clusters were from Lineage 2, 25 isolates in 6 SNP-clusters were from Lineage 3, and 83 isolates in 15 SNP-clusters were from Lineage 4. One of these SNP-clusters, the pair Mtb_4212 and Mtb_4878, was identified in NSW isolates; the two patients involved had no known direct contact, but had similar demographics and both had recently migrated to Australia from Nepal.

Out of 138 isolates that were members of a SNP-cluster, MIRU-24 profiles were identical within each SNP-cluster in all but seven instances (where only the first mismatched isolate of a second MIRU-24 cluster within a SNP-cluster was counted as an instance; see Figure 3 and Supplementary Table S2). Across these seven instances, all differed at only a single MIRU locus (424, 2163b, 3171, 3192, or 4052), the median difference at that MIRU locus was two repeats, and the median number of substitutions separating isolates with different MIRU-24 profiles was 2, indicating recent common ancestry despite different MIRU-24 profiles.

The true positive rate or sensitivity of identical MIRU-24 for detection of recent transmission (defined as <10 SNPs) was 95% (131/138, 95% CI: 90–98%), with seven instances where SNP-clustered isolates were misclassified by MIRU-24 (see Supplementary Table S2). The true negative rate or specificity of MIRU-24 was 47% (50/107, 95% CI: 38–56%). In this selected group of isolates, the prevalence of recent transmission by SNP clustering was 56% (138/245), the positive predictive value of an identical MIRU-24 for detecting recent transmission was 72% (127/177, 95% CI: 65–78%), and the negative predictive value was 84% (57/68, 95% CI: 73–91%).

The Simpson diversity index for MIRU remained reassuringly high (0.96). However, by MIRU-24, 177 isolates were clustered, vs. 138 isolates by the permissive criterion of substitution distance <10. This represents a reduction in potential secondary cases from 149 isolates in 28 clusters with MIRU, to 108 isolates in 30 clusters with SNP typing (of which 13 isolates in seven SNP clusters had the “wrong” MIRU-24). In other words, 32% (48/149) of potential secondary cases by MIRU-24 were “false positives,” using a permissive standard for recent transmission.

### MIRU Homoplasy

**Figure 4:**
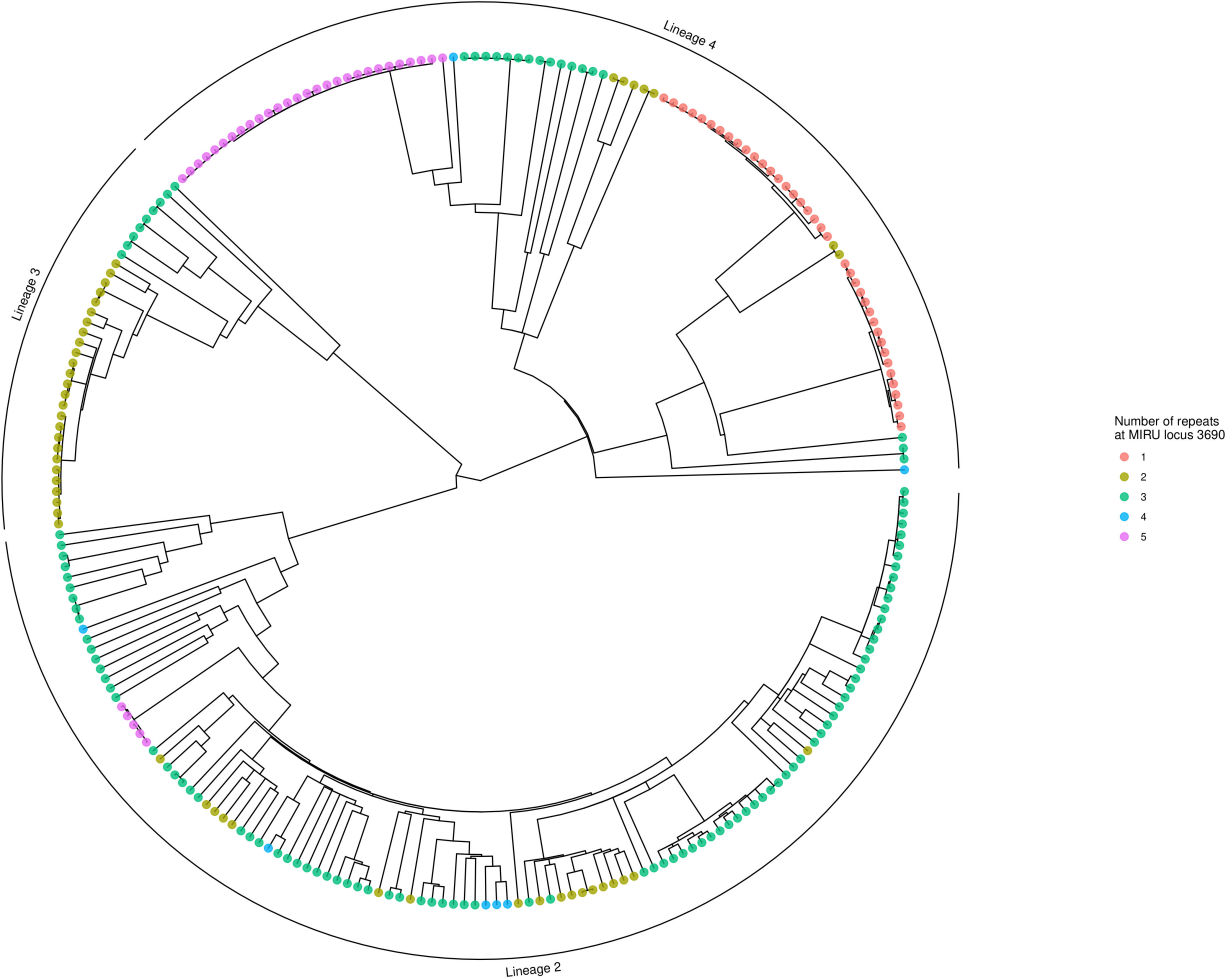
Phylogenetic tree showing number of repeats at locus 3690. The tree is identical to that shown in Figure 3, except tips are colored to designate the number of repeats at locus 3690 for each isolate. Numerous instances of homoplasy are seen in Lineages 2 and 4. The tree was generated using *R ggtree* (41).

**Figure 5:**
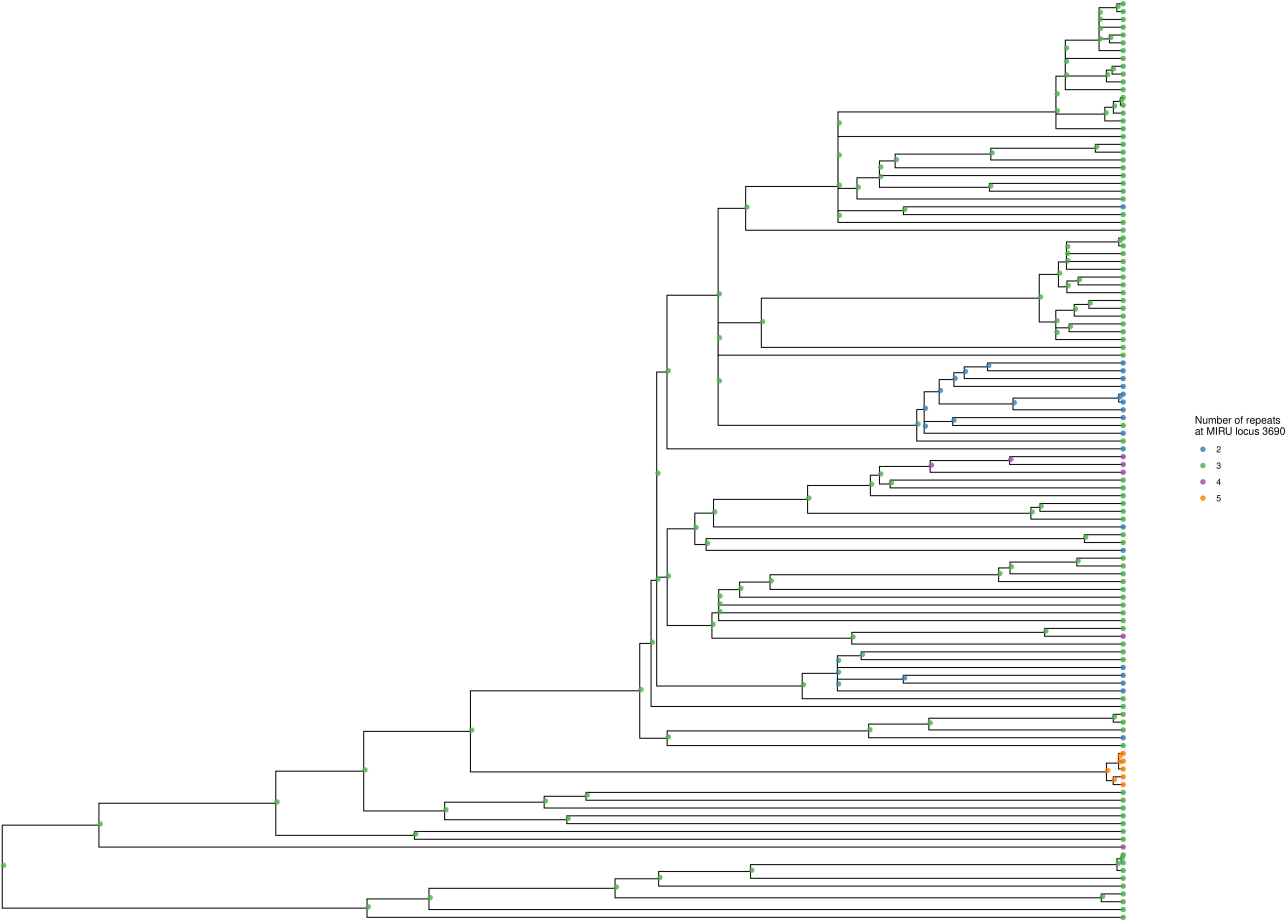
Ancestral state reconstruction for locus 3690, shown for Lineage 2 subtree. The tree corresponds to the Lineage 2 subtree from Figures 3, 4. Tips are colored to designate the number of repeats at locus 3690 for each isolate. Internal nodes show the ancestral state likelihoods, generated using the maximum likelihood method of *R phytools*::*rerootingMethod* (38), and displayed as a pie chart with the same color scheme as the tips. The tree was generated using *R ggtree* (41).

Using locus 3690 as a representative example, Figure 4 shows the number of repeats for each isolate in comparison to the phylogeny. For this locus, there is extensive homoplasy affecting lineages 2 and 4. Locus 3690 is not exceptional; all 20 loci with a retention index other than one demonstrate homoplasy. Treating the number of repeats at each MIRU locus as a discrete trait or characteristic, the *R* package *phytools* (38) was used to model the ancestral states (i.e., number of repeats) for MIRU loci using a maximum likelihood algorithm. Focusing on Lineage 2 for clarity, the most likely number of repeats at internal nodes of the tree for representative MIRU locus 3690 are displayed in Figure 5. This demonstrates at least nine instances of repeat number homoplasy at locus 3690 arising in the phylogeny of these 118 Lineage 2 isolates.

Treating the number of repeats at a MIRU locus as a continuous trait or characteristic (20, 21), the *R* package *bayou* was used to model the evolution of repeat number using a Bayesian reversible-jump MCMC model with stepping stone importance sampling to estimate marginal likelihood. This was used to compare the Brownian motion and Ornstein–Uhlenbeck models of trait evolution. MIRU-24 loci with Simpson diversity index >0.2 were individually analyzed. The simpler Brownian motion model was favored for all 16 of these loci; in other words, there was no evidence that number of repeats at any of these loci was subject to selective pressure over the branches of this phylogeny.

**Figure 6:**
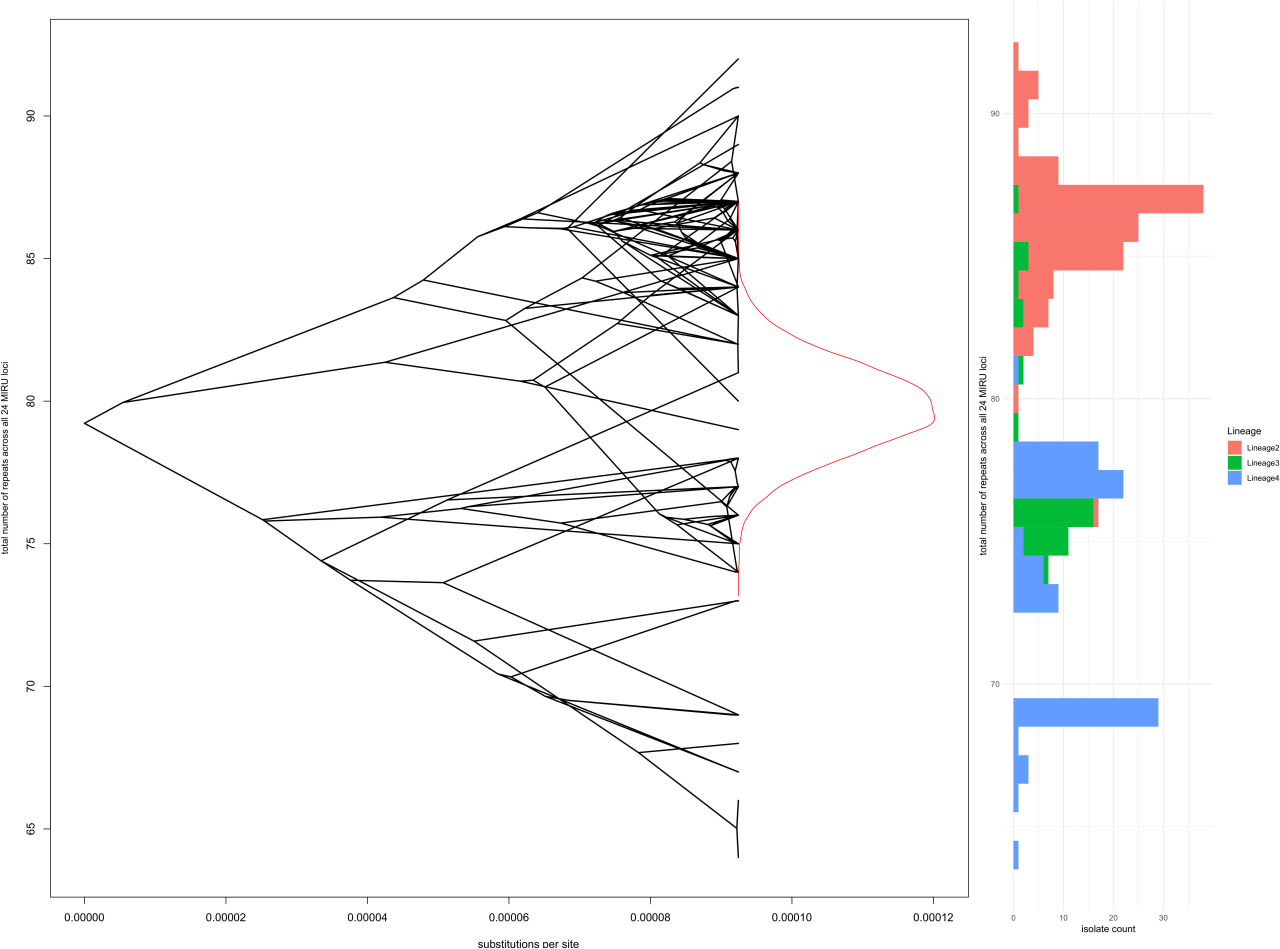
Total number of repeats for all 24 loci in comparison to whole genome phylogeny and Lineage. The left panel shows a “phenogram,” overlaying the same phylogenetic tree shown in Figures 3, 4 onto “trait space,” where the *y*-axis represents total number of repeats. Ancestral states were modeled using *R bayou* (25), with a Brownian motion model of evolution. The Brownian motion model was favored by stepping stone marginal likelihood over the corresponding Ornstein-Uhlenbeck model. The central part of the left panel shows the MCMC sampling probability density of total number of repeats at the root node (the MRCA for Lineages 2, 3, and 4). The right panel is a histogram of total number of repeats, shaded by Lineage.

One possible selective pressure acting on repeat loci is genome reduction due to gene loss, as observed for *M. tuberculosis* and *M. leprae* compared to ancestral and environmental mycobacteria. In an attempt to capture small selective forces that might operate on multiple loci to favor more or fewer repeats across the chromosome, modeling of total repeats over all 24 loci as a continuous trait was performed using *bayou*; again there was no evidence to reject the simplest Brownian motion model of stochastic changes in repeat number, without selective pressure. Figure 6 shows the whole genome phylogeny overlaid on the total number of repeats at each node (reconstructed using *bayou* for internal nodes), a density plot of reconstructed total number of repeats at the root node, and a histogram of total repeats by lineage for the 245 isolates at the tips.

## Discussion

This analysis of *M. tuberculosis* isolates of modern lineages using genome-wide phylogeny shows evidence of extensive homoplasy of repeat number at MIRU loci. Comparison of repeat loci with genomic deletions has demonstrated fewer repeats per locus in lineages that separated later from the common ancestor of modern *M. tuberculosis* (e.g., Lineages 2-4), whilst more ancient lineages (e.g., Lineage 1) have a higher average number of repeats per locus (45). This trend was not apparent in our data; in addition we found no evidence for selective pressure operating on number of repeats at individual loci or total number of repeats across 24 loci. Stochastic variation in repeat number, without subsequent natural selection, appears to have been the dominant process since the emergence of the modern *M. tuberculosis* lineages, although weaker selective pressures operating over longer time periods cannot be excluded with confidence. It is worth noting that strains from Lineage 2, the epitome of a successful modern clade of *M. tuberculosis*, had a higher total number of repeats than other modern lineages (Figure 6). Within Lineage 2, the lowest total number of repeats was found amongst the “ancient Beijing” strains.

Extending the definition of a MIRU-24 cluster to include isolates with up to one MIRU locus mismatch was not helpful for detection of recent transmission in this dataset. This was clearly demonstrated for Lineage 2 isolates, where allowing one locus mismatch produced a “super-cluster” of 50 isolates, and two mismatched loci produced a cluster of 77 isolates that was completely uninformative regarding recent transmission. One potential advantage of allowing a mismatch is that it also dilutes random homoplasious changes in repeat numbers, maintaining cluster membership; this could be valuable if the objective of MIRU-24 was classification into major clades or lineages, rather than to detect recent transmission. However, a PCR-based SNP typing scheme would be cheaper and more accurate in these scenarios.

A limitation of this analysis is underlying bias in isolate selection. Selection bias would most strongly affect the prevalence of clustering and hence the positive and negative predictive value estimates for MIRU-24 to detect recent transmission, but could also influence the analysis of homoplasy. Within the NSW and Walker datasets isolates underwent MIRU-24 routinely; however they were chosen for sequencing based on MIRU-24 properties (MIRU-24 clusters with no mismatches for the NSW isolates; up to two mismatches for the Walker isolates). In contrast, the Merker data is intended to be globally representative of Lineage 2 strains. However, it derives primarily from a culture collection at a single major reference laboratory, and the high proportion of MDR strains within the collection is typical of culturing bias in resource-limited settings. Other datasets [e.g., (46)] captured a large number of *M. tuberculosis* isolates but did not provide corresponding MIRU-24 data. In settings with a low frequency of recent transmission, or where only a small proportion of infectious patients have isolates available for molecular epidemiology, true clustering rates and the positive predictive value of MIRU-24 clustering will be correspondingly lower.

Another limitation of this analysis is the handling of potential errors in ascertainment of repeat number at MIRU loci. MIRU-24 data employed in this study were generated by reference laboratories, and have been published previously, so are likely to match or exceed real-world quality of MIRU data. The reproducibility of MIRU-24 is good (87–100%) (47–49), but each error in repeat number has potential to generate spurious homoplasy. However, it is unlikely that the same error in repeat number would co-occur in closely related isolates (as observed in these data). Any errors in MIRU ascertainment that produce a false homoplasy signal would also represent an important limitation of MIRU. Mitigation of MIRU errors would require either routine repetition of MIRU typing or relaxation of the MIRU cluster definition, which in this dataset did not retain sufficient diversity.

Our data suggest that the number of repeats at specific MIRU loci evolve at random, without evidence of selective pressure. Homoplasy of repeat numbers reduces the utility of MIRU-24. In these data, MIRU-24 showed poor specificity and modest positive and negative predictive values for the detection of recent transmission, using a permissive SNP threshold. Unless supported by independent evidence (e.g., epidemiological links), clusters detected using MIRU-24 will often be polyphyletic and unhelpful for detecting recent transmission, especially in settings with incomplete sampling, limited local transmission, or high rates of imported disease. Inclusion of additional hypervariable repeat loci (50) could help to improve specificity, but has been superseded by WGS approaches. If WGS is not yet feasible due to resource constraints, consideration should be given to the use of a hybrid scheme, for example, a small number of hypervariable loci for diversity, supplemented with an independent typing method with rare homoplasy [e.g., SNP typing (51–53) as recommended previously (16)] that has been validated in locally prevalent *M. tuberculosis* clades.

In conclusion, we found frequent homoplasy of MIRU repeats in modern *M. tuberculosis* lineages, arising through stochastic processes without natural selection. This homoplasy contributed to relatively poor specificity and low positive predictive values for the detection of recent transmission. These findings strengthen the case for transition away from MIRU-based to WGS-based methods of typing, which display much higher discrimination and rarer homoplasy, and provide important drug resistance information.

## Data Availability Statement

The datasets generated for this study can be found in the European Nucleotide Archive, PRJEB11778, PRJEB7281, and PRJEB2221.

## Ethics Statement

The studies involving human participants were reviewed and approved by Human Research Ethics Committee University of Sydney. Written informed consent for participation was not required for this study in accordance with the national legislation and the institutional requirements.

## Author Contributions

AO, BM, and VS developed the concepts for this analysis. UG collected data and initiated the analysis of NSW MRL MIRU-24 clusters. UG, PJ, NM, and QW performed laboratory procedures including MIRU-24 and whole genome sequencing for the NSW MRL isolates. GAH-C provided expertise regarding whole genome sequencing and bioinformatic analysis. AO performed bioinformatic and statistical analyses. AO and BM drafted the initial manuscript. BM and VS supervised the research. All authors participated in manuscript revision.

## Conflict of Interest

The authors declare that the research was conducted in the absence of any commercial or financial relationships that could be construed as a potential conflict of interest.
