# Supplementary material for: Extensive Homoplasy but No Evidence of Convergent Evolution of Repeat Numbers at MIRU Loci in Modern Mycobacterium tuberculosis Lineages
Source: Front Public Health. 2020 Aug 27;8:455. doi: 10.3389/fpubh.2020.00455 (PMC7481465; doi:10.3389/fpubh.2020.00455)
Supplement: Supplementary file 1 [file Table_1.PDF]

Included libraries from Merker et al. (reference 29). Available from: https://www.ebi.ac.uk/ena/data/view/PRJEB7281

FA3_1037
FA3_1367
R04_0039
49_02
57_02
178_03
474_05
575_03
679_04
683_05
685_05
709_05
711_05
966_95
967_02
1232_02
12448_03
12615_95
1312_05
1314_04
1339_07
1500_03
1511_02
1691_01
1934_03
2280_07
2389_05
2488_02
3015_05
3075_05
3297_06
3435_06
3454_10
3684_08
3811_05
3936_02
3944_06
4203_01
4276_09
4287_09
4296_09
4403_05
4484_07
4546_04
4688_09
4703_09
4769_05
4950_04
5250_07
5331_05
5569_09
5712_95
5790_04
6265_09
6844_06
7187_09
7194_05
7253_02
7424_07
7426_10
7443_10
7453_10
7454_06
7461_10
7750_01
7942_05
8038_95
8195_08
8304_09
8372_95
8378_11
8380_11
8383_11
8455_05
8654_06
8662_05
8886_01
8888_01
8889_01
9048_05
9052_05
9589_07
9700_04
9827_01
9829_08
9847_95
10107_01
10507_09
10529_05
10530_05
10721_03
10734_04
10735_04
10737_02
10836_09
11251_09

Included libraries from Walker et al. (reference 28). Available from: https://www.ebi.ac.uk/ena/data/view/PRJEB2221

ERR025833	ERR040094	ERR046851
ERR038253	ERR040097	ERR046853
ERR038262	ERR040099	ERR046897
ERR038264	ERR040102	ERR046917
ERR038265	ERR040106	ERR046920
ERR038269	ERR040107	ERR046923
ERR038271	ERR040109	ERR046924
ERR038272	ERR046732	ERR046926
ERR038273	ERR046733	ERR046928
ERR038274	ERR046736	ERR046929
ERR038275	ERR046737	ERR046930
ERR038277	ERR046738	ERR046932
ERR038282	ERR046739	ERR046946
ERR038283	ERR046741	ERR046947
ERR038284	ERR046743	ERR046949
ERR038285	ERR046744	ERR046950
ERR038286	ERR046745	ERR046951
ERR038287	ERR046746	ERR046953
ERR038288	ERR046756	ERR046963
ERR038290	ERR046759	ERR046964
ERR038291	ERR046760	ERR046965
ERR038293	ERR046761	ERR046966
ERR038294	ERR046762	ERR046967
ERR038296	ERR046763	ERR046968
ERR038298	ERR046764	ERR046969
ERR038299	ERR046765	ERR046970
ERR038300	ERR046768	ERR046971
ERR039328	ERR046775	ERR046992
ERR039329	ERR046776	ERR046993
ERR039330	ERR046781	ERR046995
ERR039331	ERR046782	ERR046996
ERR039332	ERR046783	ERR072021
ERR039333	ERR046791	ERR072024
ERR039334	ERR046793	ERR072041
ERR039339	ERR046799	ERR072042
ERR039340	ERR046819	ERR072044
ERR039341	ERR046836	ERR072045
ERR039342	ERR046838	ERR072046
ERR040086	ERR046840	ERR072047
ERR040089	ERR046841	ERR072048
ERR040091	ERR046842	ERR072050
ERR040093	ERR046845	ERR072051

ERR072065
ERR072077
ERR072080
ERR072087
ERR072094
ERR072096

Included libraries from Gurjav et al. (reference 6). Available from:
https://www.ebi.ac.uk/ena/data/view/PRJEB11778

Mtb_0058
Mtb_2043
Mtb_2213
Mtb_2400
Mtb_3138
Mtb_3578
Mtb_3715
Mtb_4212
Mtb_4277
Mtb_4302
Mtb_4541
Mtb_4616
Mtb_4628
Mtb_4690
Mtb_4804
Mtb_4805
Mtb_4878
