# Supplementary material for: Extensive Homoplasy but No Evidence of Convergent Evolution of Repeat Numbers at MIRU Loci in Modern Mycobacterium tuberculosis Lineages
Source: Front Public Health. 2020 Aug 27;8:455. doi: 10.3389/fpubh.2020.00455 (PMC7481465; doi:10.3389/fpubh.2020.00455)
Supplement: Supplementary file 2 [file Table_2.PDF]

Extensive homoplasy but no convergent evolution of repeat numbers at MIRU loci in modern *Mycobacterium tuberculosis* lineages

Alexander C. Outhred, Ulziijargal Gurjav, Peter Jelfs, Nadine McCallum, Qinning Wang, Grant A. Hill-Cawthorne, Ben J. Marais, Vitali Sintchenko

MIRU mismatches within a SNP-cluster

First isolate with non-congruent MIRU	Other isolates with same non-congruent MIRU	MIRU locus that was non-congruent	Difference in number of MIRU repeats between congruent and non-congruent MIRU within same SNP-cluster	Number of substitutions to nearest SNP-cluster isolate with congruent MIRU	Collection containing these isolates
685_05	-	3192	1	2	Merker
57_02	7750_01	4052	3	8	Merker
ERR039331	ERR039332	424	2	2	Walker
ERR038287	ERR038288	424	2	2	Walker
ERR039333	ERR039334	424	2	1	Walker
ERR039330	ERR046732	3171	2	2	Walker
ERR046851	ERR046853	2163b	1	4	Walker
**No. of instances:**	7	**Mean:**	1.9	3.0	
**No. of isolates:**	13	**Median:**	2.0	2.0	
