# Supplementary figures and images for: Extensive Homoplasy but No Evidence of Convergent Evolution of Repeat Numbers at MIRU Loci in Modern Mycobacterium tuberculosis Lineages
Source: Front Public Health. 2020 Aug 27;8:455. doi: 10.3389/fpubh.2020.00455 (PMC7481465; doi:10.3389/fpubh.2020.00455)

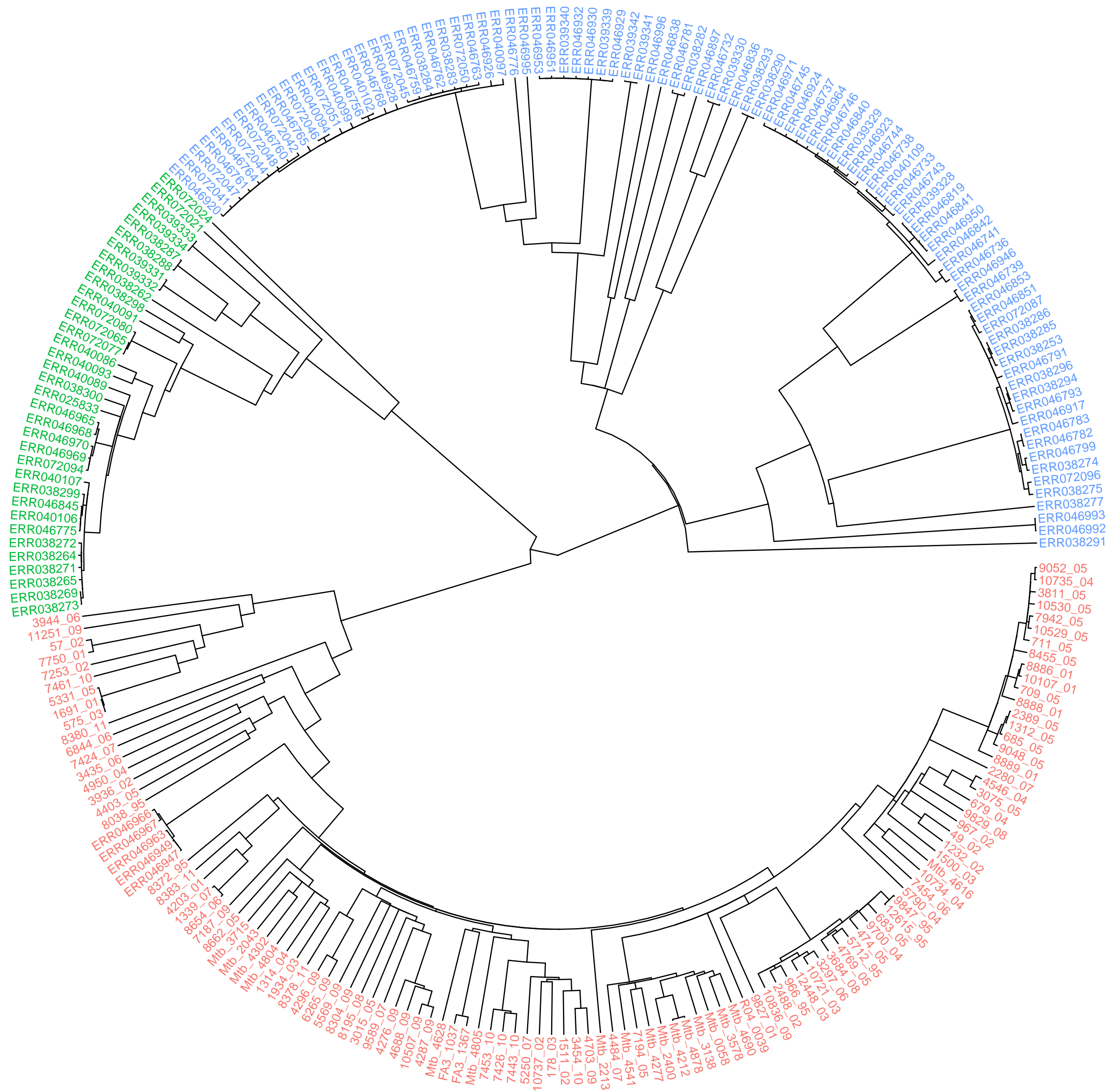

## MTBC Lineage

- a Lineage2
- a Lineage3
- a Lineage4

Supplement: Supplementary file 3 [file Image_1.PDF]

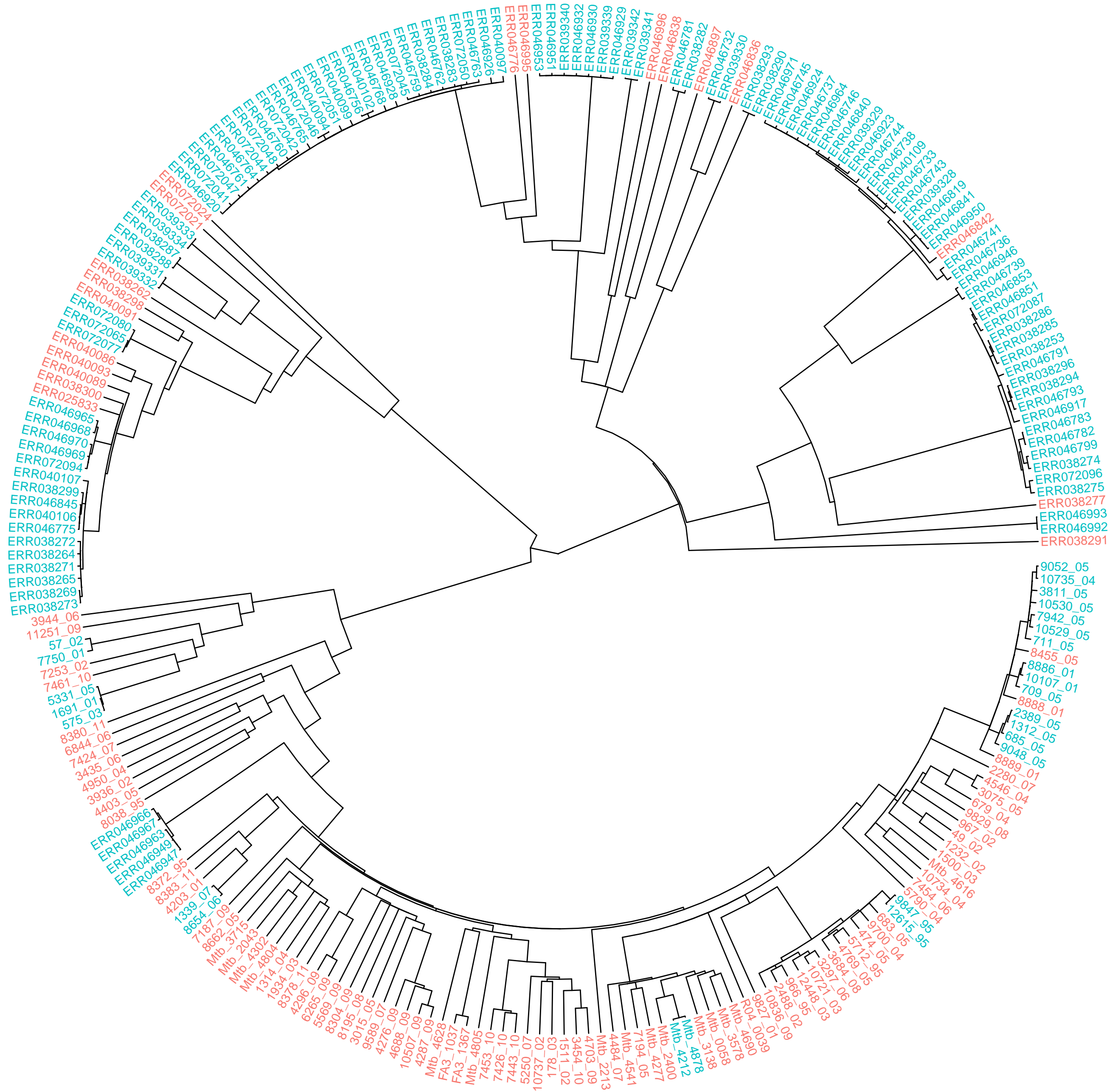

Supplement: Supplementary file 4 [file Image_2.PDF]
